# Severe neurological outcomes after very early bilateral nephrectomies in patients with autosomal recessive polycystic kidney disease (ARPKD)

**DOI:** 10.1038/s41598-020-71956-1

**Published:** 2020-09-29

**Authors:** Kathrin Burgmaier, Gema Ariceta, Martin Bald, Anja Katrin Buescher, Mathias Burgmaier, Florian Erger, Michaela Gessner, Ibrahim Gokce, Jens König, Claudia Kowalewska, Laura Massella, Antonio Mastrangelo, Djalila Mekahli, Lars Pape, Ludwig Patzer, Alexandra Potemkina, Gesa Schalk, Raphael Schild, Rukshana Shroff, Maria Szczepanska, Katarzyna Taranta-Janusz, Marcin Tkaczyk, Lutz Thorsten Weber, Elke Wühl, Donald Wurm, Simone Wygoda, Ilona Zagozdzon, Jörg Dötsch, Jun Oh, Franz Schaefer, Max Christoph Liebau, The ARegPKD consortium, Loai Akram Eid, Klaus Arbeiter, Nadejda Ranguelov, Laure Collard, Aurélie De Mul, Markus Feldkoetter, Tomas Seeman, Julia Thumfart, Franziska Grundmann, Matthias Galiano, Björn Buchholz, Rainer Buescher, Karsten Häffner, Oliver Gross, Wanja Bernhardt, Anke Doyon, Michael Henn, Jan Halbritter, Ute Derichs, Günter Klaus, Bärbel Lange-Sperandio, Barbara Uetz, Marcus Benz, Andrea Titieni, Hagen Staude, Heinz E. Leichter, Neveen A. Soliman, Luis Enrique Lara, Francisco de la Cerda Ojeda, Jerome Harambat, Bruno Ranchin, Marc Fila, Claire Dossier, Olivia Boyer, Matko Marlais, Stella Stabouli, Nakysa Hooman, Francesca Mencarelli, William Morello, Germana Longo, Francesco Emma, Dovile Ruzgiene, Anna Wasilewska, Irena Balasz-Chmielewska, Monika Miklaszewska, Malgorzata Stanczyk, Przemyslaw Sikora, Mieczyslaw Litwin, Aurelia Morawiec-Knysak, Ana Teixeira, Gordana Milosevski-Lomic, Larisa Prikhodina, Rina Rus, Houweyda Jilani, Engin Melek, Ali Duzova, Alper Soylu, Cengiz Candan, Lale Sever, Alev Yilmaz, Neslihan Cicek, Nurver Akinci, Sevgi Mir, Ismail Dursun, Yilmaz Tabel, Hulya Nalcacioglu

**Affiliations:** 1 Department of Pediatrics, Faculty of Medicine, University Hospital Cologne and University of Cologne, Kerpener Str. 62, 50937 Cologne, Germany; 2 Department of Pediatric Nephrology, University Hospital Vall d’Hebron, Barcelona, Spain; 3 Department of Pediatric Nephrology, Klinikum Stuttgart, Olga Children’s Hospital, Stuttgart, Germany; 4 Department of Pediatrics II, University Hospital Essen, Essen, Germany; 5 Department of Internal Medicine I, University Hospital of the RWTH Aachen, Aachen, Germany; 6 Institute of Human Genetics, University Hospital of Cologne, Cologne, Germany; 7 Center for Molecular Medicine Cologne, University of Cologne, Faculty of Medicine and University Hospital Cologne, Cologne, Germany; 8 Department of General Pediatrics and Hematology/Oncology, Children’s University Hospital Tuebingen, Tuebingen, Germany; 9 Division of Pediatric Nephrology, Research and Training Hospital, Marmara University, Istanbul, Turkey; 10 Department of General Pediatrics, University Hospital Muenster, Muenster, Germany; 11 The Children’s Memorial Health Institute, Warsaw, Poland; 12 Division of Nephrology, Department of Pediatric Subspecialties, Bambino Gesù Children’s Hospital, IRCCS, Rome, Italy; 13 Pediatric Nephrology, Dialysis and Transplant Unit, Fondazione IRCCS Cà Granda, Ospedale Maggiore Policlinico, Milan, Italy; 14 Department of Development and Regeneration, PKD Research Group, KU Leuven, Leuven, Belgium; 15 Department of Pediatric Nephrology, University Hospitals Leuven, Leuven, Belgium; 16 Department of Pediatric Kidney, Liver and Metabolic Diseases, Hannover Medical School, Hannover, Germany; 17 Children’s Hospital St. Elisabeth and St. Barbara, Halle (Saale), Germany; 18 Department of Paediatrics and Adolescent Medicine, Medical University Vienna, Vienna, Austria; 19 Department of Pediatrics, University Hospital Bonn, Bonn, Germany; 20 University Children’s Hospital, University Medical Center Hamburg Eppendorf, Hamburg, Germany; 21 UCL Great Ormond Street Hospital for Children Institute of Child Health, UCL, London, UK; 22 Department of Pediatrics, Faculty of Medical Sciences in Zabrze, SUM in Katowice, Katowice, Poland; 23 Department of Paediatrics and Nephrology, Medical University of Bialystok, Bialystok, Poland; 24 Department of Pediatrics, Immunology and Nephrology, Polish Mother’s Memorial Hospital Research Institute, Lodz, Poland; 25 Division of Pediatric Nephrology, Center for Pediatrics and Adolescent Medicine, University Hospital Heidelberg, Heidelberg, Germany; 26 Department of Pediatrics, Klinikum Saarbrücken, Saarbrücken, Germany; 27 Clinic for Children and Adolescents, Hospital St. Georg, Leipzig, Germany; 28 Department of Pediatrics, Nephrology and Hypertension, Medical University of Gdansk, Gdansk, Poland; 29 Pediatric Nephrology Department, Dubai Kidney Center of Excellence, Dubai Hospital, Dubai, UAE; 30 Department of Pediatrics, Université Catholique de Louvain Medical School, Saint-Luc Academic Hospital, Brussels, Belgium; 31 Centre de référence de Néphrologie Pédiatrique Sud, Clinique de l’Espérance, Montegnee, Belgium; 32 Hôpitaux Universtaires de Genève, Unité Romande de Néphrologie Pédiatrique, Geneva, Switzerland; 33 Pediatric Nephrology Unit, University Children’s Hospital, Zurich, Switzerland; 34 Department of Pediatrics, University Hospital Motol, 2nd Faculty of Medicine, Charles University Prague, Prague, Czech Republic; 35 Department of Pediatric Nephrology, Charité–Universitätsmedizin Berlin, Corporate Member of Freie Universität Berlin, Humboldt-Universität zu Berlin, and Berlin Institute of Health, Berlin, Germany; 36 Department II of Internal Medicine, University Hospital of Cologne, Cologne, Germany; 37 Department of Pediatrics and Adolescent Medicine, University of Erlangen-Nürnberg (FAU), Erlangen, Germany; 38 Department of Nephrology and Hypertension, University of Erlangen-Nürnberg, Erlangen, Germany; 39 Department of General Pediatrics, Adolescent Medicine and Neonatology, Freiburg University Hospital, Freiburg, Germany; 40 Clinic for Nephrology and Rheumatology, University Medical Center Goettingen, Goettingen, Germany; 41 Nephrology Clinic Hannover, Hannover, Germany; 42 Division of Nephrology, University Hospital Leipzig, Leipzig, Germany; 43 Pediatric Nephrology, Center for Paediatric and Adolescent Medicine, University Medical Clinic, Mainz, Germany; 44 KfH Center of Paediatric Nephrology, University Hospital of Marburg, Marburg, Germany; 45 Dr. von Haunersches Kinderspital, Ludwigs Maximilian University, Munich, Germany; 46 KfH Center of Pediatric Nephrology, Children’s Hospital Munich Schwabing, Munich, Germany; 47 Klinik für Kinder-und Jugendmedizin, Klinikum Dritter Orden, München, Germany; 48 Pediatric Nephrology, University Children’s Hospital Rostock, Rostock, Germany; 49 Olga Children’s Hospital, Clinic Stuttgart, Stuttgart, Germany; 50 Department of Pediatrics, Center of Pediatric Nephrology and Transplantation, Kasr Al Ainy School of Medicine, Cairo University, Cairo, Egypt; 51 Hospital Virgen del Rocío, Sevilla, Spain; 52 Department of Pediatrics, Bordeaux University Hospital, Bordeaux, France; 53 Pediatric Nephrology Unit, Hôpital Femme Mere Enfant, Hospices Civils de Lyon, Lyon, France; 54 Pediatric Nephrology Unit, CHU Arnaud de Villeneuve-Université de Montpellier, Montpellier, France; 55 Service de Néphrologie Pédiatrique, Hôpital Robert-debré, Paris, France; 56 Department of Pediatric Nephrology and Kidney Transplantation, Necker Hospital, Paris, France; 57 First Department of Pediatrics, Hippokration Hospital, Aristotle University of Thessaloniki, Thessaloniki, Greece; 58 Department of Pediatric Nephrology, Ali-Asghar Children Hospital, University of Medical Sciences, Tehran, Iran; 59 Nephrology and Dialysis Unit, Department of Pediatrics, Azienda Ospedaliero Universitaria Sant’Orsola-Malpighi, Bologna, Italy; 60 Nephrology, Dialysis and Transplant Unit, Department of Women’s and Children’s Health, University Hospital of Padova, Padova, Italy; 61 Clinic of Children Diseases, Institute of Clinical Medicine, Vilnius University, Vilnius, Lithuania; 62 Department of Pediatric Nephrology and Hypertension, Faculty of Medicine, Jagiellonian University Medical College, Kraków, Poland; 63 Department of Pediatric Nephrology, Medical University of Lublin, Lublin, Poland; 64 Pediatric Nephrology, Centro Hospitalar São João, Porto, Portugal; 65 Department of Nephrology, University Children’s Hospital, Belgrade, Serbia; 66 Department of Inherited and Acquired Kidney Diseases, Research Clinical Institute for Pediatrics, Pirogov Russian National Research Medical University, Moscow, Russia; 67 Department of Paediatric Nephrology, University Children’s Hospital, University Medical Centre Ljubljana, Ljubljana, Slovenia; 68 Service des Maladies Congénitales et Héréditaires, CHU Mongi Slim La Marsa, Sidi Daoud La Marsa, Tunis, Tunisia; 69 Department of Pediatric Nephrology, Faculty of Medicine, Cukurova University, Adana, Turkey; 70 Department of Pediatrics, Division of Pediatric Nephrology, Faculty of Medicine, Hacettepe University, Ankara, Turkey; 71 Department of Pediatric Nephrology, Dokuz Eylul University Medical Faculty, Balcova, Izmir, Turkey; 72 Division of Pediatric Nephrology, Istanbul Medeniyet University, Göztepe Hospital, Istanbul, Turkey; 73 Department of Pediatric Nephrology, Cerrahpaşa School of Medicine, Istanbul University, Istanbul, Turkey; 74 Pediatric Nephrology Department, Istanbul Faculty of Medicine, Istanbul University, Istanbul, Turkey; 75 Division of Pediatric Nephrology, Sisli Etfal Training and Research Hospital, Istanbul, Turkey; 76 Department of Pediatric Nephrology, Ege University Medical Faculty, Izmir, Turkey; 77 Department of Pediatric Nephrology, Faculty of Medicine, Erciyes University, Kayseri, Turkey; 78 Department of Pediatric Nephrology, Faculty of Medicine, İnönü University, Malatya, Turkey; 79 Division of Pediatric Nephrology, Faculty of Medicine, Ondokuz Mayis University, Samsun, Turkey

**Keywords:** Paediatric kidney disease, Polycystic kidney disease

## Abstract

To test the association between bilateral nephrectomies in patients with autosomal recessive polycystic kidney disease (ARPKD) and long-term clinical outcome and to identify risk factors for severe outcomes, a dataset comprising 504 patients from the international registry study ARegPKD was analyzed for characteristics and complications of patients with very early (≤ 3 months; VEBNE) and early (4–15 months; EBNE) bilateral nephrectomies. Patients with very early dialysis (VED, onset ≤ 3 months) without bilateral nephrectomies and patients with total kidney volumes (TKV) comparable to VEBNE infants served as additional control groups. We identified 19 children with VEBNE, 9 with EBNE, 12 with VED and 11 in the TKV control group. VEBNE patients suffered more frequently from severe neurological complications in comparison to all control patients. Very early bilateral nephrectomies and documentation of severe hypotensive episodes were independent risk factors for severe neurological complications. Bilateral nephrectomies within the first 3 months of life are associated with a risk of severe neurological complications later in life. Our data support a very cautious indication of very early bilateral nephrectomies in ARPKD, especially in patients with residual kidney function, and emphasize the importance of avoiding severe hypotensive episodes in this at-risk cohort.

## Introduction

Autosomal recessive polycystic kidney disease (ARPKD) is a severe and mostly early-onset hepatorenal disorder mainly caused by mutations in the *PKHD1* gene^1–3^. Mutations in a second gene, *DZIP1L*, have recently been associated with an ARPKD phenotype^3,4^. Affected newborns may suffer from massive kidney enlargement and may require early kidney replacement therapy (KRT). Peritoneal dialysis (PD) is then considered the dialysis modality of choice^5^. It has been suggested that large kidneys may contribute to respiratory problems and may hamper nutritional support^6,7^. The respiratory situation in ARPKD neonates can be challenging due to pulmonary hypoplasia and additional abdominal pressure by PD. Furthermore, concerns about the feasibility of PD in cases of massive renal enlargement have been raised. In some infants with severe ARPKD, uni- or bilateral nephrectomies are therefore performed with the aim to improve the respiratory, nutritional and dialysis conditions^6–8^. However, nephrectomies have not been shown to improve the respiratory situation and the rationale for nutritional improvement is based on small studies regarding unilateral nephrectomy^9–11^. Even in severely affected infants, residual kidney function may be preserved in ARPKD and may even improve during the first months of life^12^. Obviously, bilateral nephrectomies result in a life-long need for KRT. Additionally, arterial hypotension has been reported after bilateral nephrectomies and may contribute to neurological or ophthalmological complications in young PD patients^13–16^. The association of early bilateral nephrectomies in general and of the timing of the second nephrectomy in particular with long-term outcome in ARPKD patients remains unclear. To address this topic, we analyzed the comprehensive pre-, peri- and postnatal information captured in the ARegPKD registry study^17,18^.

## Results

### Characteristics of patient subgroups

At the timepoint of analysis, 504 patients with the clinical diagnosis of ARPKD and sufficient data quality were registered in the ARegPKD registry. Of these, 19 patients (3.8%) with very early bilateral nephrectomies (within first 3 months of life, VEBNE) and further 9 patients (1.8%) with early bilateral nephrectomies (first nephrectomy within first 15 months of life, second nephrectomy at age 4–15 months, EBNE) were identified. We included a control group of 11 patients, who had very large total kidney volumes (TKV) comparable to VEBNE patients (TKV >  ~ 200 ml, measured by ultrasound within the first 3 months of life), but were not treated with early nephrectomy or dialysis (TKV control group). Pole-to-pole lengths, single kidney volumes and total kidney volumes did not differ significantly between VEBNE and TKV control patients but TKV control patients were slightly older at measurement (Table 1). To account for effects of very early dialysis (VED) onset, an additional control group was defined with 12 ARPKD patients with dialysis onset within the first 3 months of life without bilateral nephrectomies in their disease course (VED). Patients were grouped according to their clinical status at time of last documented observation in the registry or at time prior to their death. The patient characteristics regarding nephrectomies and KRT are depicted in Table 2.

**Table 1:** Sonographic kidney measurements in patients with very early bilateral nephrectomies and patients from the TKV control group, kidney sonography performed within the first 3 months of life.

	VEBNE n = 19	TKV Control group n = 11	*P*
Age at kidney sonography, n Mean (SD), mo Median (IQR), mo	16/19 0.7 (0.4) 0.6 (0.3–1.0)	11/11 1.5 (0.9) 1.5 (0.6–2.4)	0.009
PTP left kidney, n Mean (SD), cm Median (IQR), cm SDS, n Mean (SD) Median (IQR)	7/19 9.6 (1.5) 10.0 (9.2–10.5) 4/19 20.1 (2.1) 20.6 (17.8–21.8)	8/11 9.7 (1.2) 10.0 (8.5–10.7) 8/11 22.9 (3.9) 24.0 (18.9–26.6)	0.96 0.28
PTP right kidney, n Mean (SD), cm Median (IQR), cm SDS, n Mean (SD) Median (IQR)	6/19 9.8 (1.6) 10.4 (9.1–10.6) 4/19 19.7 (4.7) 19.0 (15.7–24.4)	8/11 9.6 (0.8) 9.5 (9.2–10.1) 8/11 22.7 (2.8) 23.0 (20.0–24.6)	0.23 0.28
Volume left kidney, n Mean (SD), ml Median (IQR), ml SDS, n Mean (SD) Median (IQR)	11/19 188.3 (84.7) 208.0 (105.0–260.0) 10/19 50.2 (25.3) 59.3 (22.6–66.7)	11/11 138.6 (64.8) 105.0 (96.0–162.0) 9/11 39.9 (17.9) 32.7 (24.6–49.0)	0.15 0.50
Volume right kidney, n Mean (SD), ml Median (IQR), ml SDS, n Mean (SD) Median (IQR)	11/19 189.5 (79.3) 217.0 (103.0–250.0) 9/19 48.6 (24.1) 48.8 (23.4–68.0)	11/11 156.0 (53.6) 144.0 (119.0–173.0) 9/11 44.5 (14.4) 45.5 (32.9–52.9)	0.48 0.86
Total kidney volume, n Mean (SD), ml Median (IQR), ml Min–max, ml	9/19 369.9 (176.9) 437.0 (205.0–535.0) 130–580	11/11 294.6 (116.2) 257.0 (217.0–335.0) 194–580	0.60

Continuous variables are expressed as mean (± SD) and median (IQR). *P* values (right column) were derived using Mann–Whitney U tests.

*PTP* pole-to-pole, *SDS* standard deviation score.

**Table 2:** Characteristics of patient subgroups regarding nephrectomies and kidney replacement therapy.

	VEBNE n = 19		EBNE n = 9		VED n = 12	TKV control n = 11
Age at 1st NE, n	19		9		2	2
Mean (SD), mo	1.0 (0.8)		4.1 (2.0)		1.8 (0.3)	75.3 (44.8)
Median (IQR or min;max), mo	0.8 (0.4–1.3)		4.2 (2.7–6.0)		1.8 (1.6;2.0)	75.3 (43.6;107.0)
Age at 2nd NE, n	19		9		–	–
Mean (SD), mo	1.5 (0.8)		7.4 (3.7)			
Median (IQR), mo	1.6 (0.7–2.0)		5.9 (4.5–11.5)			
Age at simultaneous bilateral NE, n	2		–		–	–
Mean (SD), mo	2.6 (0.6)					
Median (IQR or min;max), mo	2.6 (2.2;3.0)					
Localisation 1st NE right, n (%)	9/17 (53%)		4/9 (44%)		1/2 (50%)	2/2 (100%)
Indications for NE (multiple answering possible)	1st	2nd	1st	2nd	1st	1st
Respiratory failure/ventilation (problems), n (%)	7/19 (37%)	4/19 (21%)	4/9 (44%)	2/9 (22%)	1/2 (50%)	–
Nutritional problems/FTT, n (%)	1/19 (5%)	1/19 (5%)	0/9 (0%)	2/9 (22%)	–	–
Arterial hypertension, n (%)	1/19 (5%)	1/19 (5%)	2/9 (22%)	2/9 (22%)	–	–
Onset of PD, n (%)	8/19 (42%)	3/19 (16%)	0/9 (0%)	0/9 (0%)	–	–
PD problems/inefficacy, n (%)	1/19 (5%)	5/19 (26%)	1/9 (11%)	1/9 (11%)	–	–
Abdominal distension/kidney enlargement, n (%)	7/19 (37%)	7/19 (37%)	1/9 (11%)	1/9 (11%)	–	–
Massive growth of kidney after 1st NE, n (%)	–	3/19 (16%)	–	0/9 (0%)	–	–
Indication unknown, n (%)	2/19 (11%)	4/19 (21%)	2/9 (22%)	3/9 (33%)	1/2 (50%)	–
Others, n (%)	–	–	–	–	–	2/2 (100%) CLKTx
Age at onset of dialysis/KRT, n	19		7		12	2
Mean (SD), mo	0.8 (0.8)		5.4 (4.1)		1.1 (1.0)	72.0 (49.6)
Median (IQR or min;max), mo	0.6 (0.2–1.2)		5.0 (1.8–8.1)		0.6 (0.4–1.9)	72.0 (37.0;107.0)
Type of dialysis/KRT	PD 16/19, HD 1/19, CVVH 2/19		PD 5/7, HD 2/7		PD 10/12, HD 1/12, CVVH 1/12	HD 1/2, CLKTx 1/2
Onset of dialysis prior to 1st NE, n (%)	7/19 (37%) (5 PD, 2 CVVH)		2/7 (29%) (1 PD, 1 HD)		No NE at all: 10/12 (83%) (9 PD, 1 CVVH)	1/2 (50%) (1 HD)
Onset of dialysis after 1st NE, n (%)	11/19 (58%) (10 PD, 1 HD)		3/7 (43%) (3 PD)		2/12 (17%) (1 PD, 1 CVVH)	–
Onset of dialysis after 2nd NE, n (%)	1/19 (5%) (1 PD)		2/7 (29%) (1 PD, 1 HD)		–	–

Categorical values given as n/n total (percentage); continuous variables, as mean ± SD and median (IQR or min;max) with min;max indicated in case of n ≤ 3.

*CLKTx* combined liver and kidney transplantation, *CVVH* continuous veno-venous hemofiltration, *EBNE* early bilateral nephrectomies, *FTT* failure to thrive, *HD* hemodialysis, *KRT* kidney replacement therapy, *NE* nephrectomy, *PD* peritoneal dialysis, *TKV* total kidney volume, *VEBNE* very early bilateral nephrectomies, *VED* very early dialysis.

### Indications for nephrectomies

In VEBNE patients, indications for nephrectomies encompassed respiratory failure and/or ventilation problems, abdominal distension and/or massive kidney enlargement and onset of or problems with PD (Table 2). Most patients with sequential nephrectomies started dialysis after the first nephrectomy (11/17, 65%). Two patients of the VED group received very early unilateral nephrectomy. In the TKV control group, two patients received unilateral nephrectomy during combined liver and kidney transplantation surgery later in life.

### Pre- and perinatal characteristics

Detailed information on patient characteristics is provided in Table 3. VEBNE and TKV control patients more frequently showed poor postnatal respiratory adaptation and were more often admitted to neonatal intensive care units (NICU). Other characteristics—including gestational age, birth weight, length, head circumference as well as Apgar scores and requirement of respiratory support—did not show relevant differences between the groups.

**Table 3:** Pre-, peri- and postnatal information by patient group.

	VEBNE n = 19	EBNE n = 9	VED n = 12	TKV control n = 11	*P*
Sex (male), n (%)	8/19 (42%)	5/9 (56%)	6/12 (50%)	5/11 (46%)	0.92
**Prenatal anomalies, n (%)**	19/19 (100%)	7/9 (78%)	11/12 (92%)	10/10 (100%)	0.10
Oligo- or anhydramnios, n (%)	17/18 (94%)	6/9 (67%)	10/12 (83%)	10/10 (100%)	0.11
Gw at diagnosis, n	15	6	9	9	0.12
Mean (SD)	27.2 (3.8)	31.8 (4.1)	30.9 (5.6)	29.1 (5.9)	
Median (IQR)	27.0 (24.0–30.0)	31.5 (28.0–34.5)	31.0 (27.0–36.0)	29.0 (25.0–34.0)	
Increased echogenicity, n (%)	10/13 (77%)	3/7 (43%)	5/9 (56%)	8/8 (100%)	0.07
Gw at diagnosis, n	9	3	5	7	0.70
Mean (SD)	28.9 (2.4)	27.7 (5.5)	30.6 (6.5)	28.7 (6.7)	
Median (IQR or min;max)	28.0 (27.5–30.5)	28.0 (22.0;33.0)	32.0 (25.0–35.5)	29.0 (24.0–36.0)	
Renal hyperplasia, n (%)	13/16 (81%)	4/8 (50%)	6/9 (67%)	7/7 (100%)	0.13
Renal cysts, n (%)	13/16 (81%)	5/9 (56%)	7/11 (64%)	7/7 (100%)	0.16
**Perinatal information**					
Normal vaginal delivery, n (%)	5/18 (28%)	4/9 (44%)	9/11 (82%)**^§^	3/10 (30%)	0.03
Perinatal problems, n (%)	17/19 (90%)	7/9 (78%)	7/12 (58%)	10/10 (100%)	0.06
NICU, n (%)	17/19 (90%)	6/9 (67%)	7/12 (58%)	10/10 (100%)^#˦^	0.04
NICU days, n	15	6	7	9	0.06
Mean (SD)	60 (35)	131 (207)	91 (134)	30 (52)	
Median (IQR)	50 (35–85)	39 (14–248)	45 (20–83)	7 (3–39)	
Poor postnatal adaptation, n (%)	16/19 (84%)^##^	3/9 (33%)	7/12 (58%)	9/10 (90%)^#^	0.02
Assisted breathing, n (%)	17/19 (90%)	7/9 (78%)	7/12 (58%)	9/10 (90%)	0.16
Pulmonary Hypertension, n (%)	8/17 (47%)	2/7 (29%)	3/11 (27%)	3/9 (33%)	0.69
**Postnatal information**					
Gestational age at birth, n	17	9	10	10	0.31
Mean (SD), weeks	35.0 (1.7)	35.9 (2.6)	36.2 (3.1)	34.8 (2.5)	
Median (IQR), weeks	35.0 (34.0–36.0)	36.0 (33.5–38.5)	37.0 (34.8–38.3)	35.0 (32.8–37.3)	
Birth weight, n	18	7	9	10	0.46
Mean (SD), kg	3.01 (0.67)	3.19 (0.62)	3.07 (0.84)	2.70 (0.57)	
Median (IQR), kg	3.00 (2.61–3.55)	3.18 (2.71–3.54)	3.41 (2.46–3.60)	2.70 (2.19–3.22)	
Birth length, n	14	6	7	9	0.52
Mean (SD), cm	47.8 (4.1)	48.5 (2.8)	49.1 (5.9)	46.3 (3.4)	
Median (IQR), cm	49.0 (45.3–51.0)	50.0 (45.5–50.3)	49.0 (45.0–52.0)	45.0 (43.3–49.5)	
Head circumference, n	14	4	8	8	0.98
Mean (SD), cm	33.0 (1.9)	33.0 (1.8)	33.4 (2.4)	32.6 (2.1)	
Median (IQR), cm	33.3 (32.4–34.5)	33.0 (31.3–34.8)	33.3 (32.0–34.6)	32.0 (31.0–35.0)	
Head circumference percentile, n	13	4	7	8	0.42
Mean (SD)	68 (26)	58 (38)	53 (30)	76 (14)	
Median (IQR)	73 (54–90)	74 (18–82)	55 (26–76)	82 (61–88)	
Microcephaly at birth (< P3), n (%)	0/14 (0%)	1/4 (25.0%)	1/8 (13%)	0/8 (0%)	0.20
Apgar 1, n	14	6	9	10	0.37
Mean (SD)	4.6 (2.3)	5.5 (3.0)	6.1 (2.4)	6.1 (2.4)	
Median	5.0 (3.0–6.3)	5.5 (3.3–8.3)	6.0 (5.0–8.0)	6.0 (5.0–8.3)	
Apgar 5, n	14	6	8	10	0.29
Mean (SD)	6.1 (2.0)	6.0 (2.2)	7.0 (2.3)	7.1 (2.1)	
Median	6.5 (5.0–8.0)	5.5 (4.5–8.3)	7.5 (6.3–8.8)	8.0 (6.0–8.3)	
Apgar 10, n	13	5	8	10	0.30
Mean (SD)	7.5 (1.3)	7.8 (1.3)	7.8 (1.7)	8.2 (2.0)	
Median	8.0 (7.5–8.0)	8.0 (6.5–9.0)	8.0 (7.3–9.0)	9.0 (7.8–9.3)	
**Genetic information**					
Variant detection in *PKHD1* n (%)	20/22 (91%) (n = 11)	5/10 (50%) (n = 5)	8/12 (67%) (n = 6)	14/14 (100%) (n = 7)	
Two truncating *PKHD1* variants, n (%)	3/11 (27%)	0/5 (0%)	1/6 (17%)	2/7 (29%)	

Binary or categorical values given as n/n total (percentage); continuous variables, as mean ± SD and median (IQR or min;max) with min;max indicated in case of n ≤ 3. *P* values (right column) were derived using Kruskal–Wallis or chi-squared tests.

*EBNE* early bilateral nephrectomies, *Gw* gestational week, *NICU* neonatal intensive care unit, *P* Percentile, *TKV* total kidney volume, *VEBNE* very early bilateral nephrectomies, *VED* very early dialysis.

**P < 0.01 for comparison with VEBNE; ^#^*P* < 0.05 for comparison with EBNE; ^##^*P* < 0.01 for comparison with EBNE; ^˦^*P* < 0.05 for comparison with VED; ^§^*P* < 0.05 for comparison with TKV control.

### Long-term complications with focus on neurological development and risk factors for severe neurological complications

In a first step, we addressed the occurrence of relevant symptoms or complications including blood pressure alterations, neurological symptoms, sepsis, cardiopulmonary resuscitation and death in a descriptive manner in the four different patient groups. Among all complications investigated, the most profound differences were observed in the neurological outcome: seizures and severe neurological complications encompassing ischemic defects, hypoxic brain damage, brain infarct, parenchymal defect, severe neurodevelopmental disorder and optic neuropathy with vision loss occurred more frequently in VEBNE patients. While 12/19 (63%) VEBNE patients were affected by severe neurological complications, this was true for only 2/9 (22%) EBNE, 2/12 (17%) VED and none of the 11 TKV control patients. Of the 12 affected VEBNE patients, three suffered from cerebral ischemia, three from infarction, bleeding or parenchymal defects, three from optic neuropathy (with vision loss), two from combined cerebral ischemia or hypoxemia and optic neuropathy, and one from cerebral ischemia with bleeding. The two EBNE patients developed sinus vein thrombosis and bilateral cerebral infarctions, the two VED patients developed posthypoxic ischemic lesions after a very difficult postnatal adaptation and hydrocephalus with brain atrophy. Neurodevelopmental delay occurred most frequently in VEBNE patients. In a re-survey with specific questions on the psychomotoric development, only one of 17 (6%) studied VEBNE patients was classified to have a normal development compared to 2/8 (25%) EBNE patients, 5/11 (46%) VED patients and 5/8 (63%) TKV control patients. Approximately one third of VEBNE patients suffered from severely or very severely affected psychomotoric development each. Younger ages at the timepoint of second nephrectomy were more frequently documented in those patients with severe neurological and developmental complications, especially timepoints of second nephrectomy within the first 3 months of life (Suppl. Figure S1). The proportions of patients receiving some form of specific therapies for developmental complications were high in all four patient groups, ranging from 55% in VED to 94% in VEBNE patients. Microcephaly was hardly found at birth but was most frequently documented in VEBNE patients in the further course (11/19, 58%). Severe hyper- and hypotensive episodes as well as cardiopulmonary resuscitation (CPR) were reported for subcohorts of VEBNE and EBNE patients, without reports in VED and TKV control patients. Proportions of patients with a septic episode prior to the evaluated neurological complication did not differ significantly between the groups (Table 4).

**Table 4:** Follow-up-complications by patient group.

	VEBNE n = 19	EBNE n = 9	VED n = 12	TKV control n = 11	*P*
**Follow-up—complications**					
Follow-up time, mo, median (IQR)	78.0 (27.0–106.0)	99.0 (84.0–156.0)	43.0 (13.0–56.0)	59.0 (32.0–106.0)	0.06
Neurodevelopmental delay, n (%)	17/19 (90%)^˦§§§^	7/9 (78%)^§^	6/12 (50%)	3/11 (27%)	0.003
Specific therapies, n (%)	15/16 (94%)	6/8 (75%)	6/11 (55%)	7/8 (88%)	0.09
Physiotherapy, n (%)	14/16 (88%)	6/8 (75%)	6/11 (55%)	4/8 (50%)	0.16
Speech therapy, n (%)	10/16 (63%)^˦˦^	4/8 (50%)^˦˦^	0/11 (0%)	2/8 (25%)	0.007
Ergotherapy, n (%)	10/16 (63%)	4/7 (57%)	2/11 (18%)	3/7 (43%)	0.14
Special teacher, n (%)	6/15 (40%)	2/8 (25%)	0/11 (0%)	1/7 (14%)	0.10
Regular school, n (%)	3/9 (33%)	2/5 (40%)	1/1 (100%)	4/4 (100%)	0.10
Psychomotoric development	n = 17	n = 8	n = 11	n = 8	
Normal, n (%)	1/17 (6%)	2/8 (25%)	5/11 (46%)*	5/8 (63%)**	0.02
Mild disorder, n (%)	4/17 (24%)	4/8 (50%)	4/11 (36%)	3/8 (38%)	0.61
Severe disorder, n (%)	6/17 (35%)^˦^	1/8 (13%)	0/11 (0%)	0/8 (0%)	0.04
Very severe disorder, n (%)	6/17 (35%)	1/8 (13%)	2/11 (18%)	0/8 (0%)	0.20
Seizures, n (%)	14/19 (74%)^#˦§^	2/9 (22%)	4/12 (33%)	3/11 (27%)	0.02
Severe neurological complication, n (%)	12/19 (63%)^#˦§§^	2/9 (22%)	2/12 (17%)	0/11 (0%)	0.001
Documentation of microcephaly (< P3), n (%)	11/19 (58%)^§§^	2/9 (22%)	4/12 (33%)^§^	0/11 (0%)	0.01
Age at 1st report, n	10	2	3	–	0.64
Mean (SD), mo	42.1 (51.0)	15.5 (13.4)	0.9 (1.0)		
Median (IQR or min;max), mo	19.5 (2.8–86.4)	15.5 (6.0; 25.0)	0.4 (0.2;2.0)		
Severe hypertensive episodes, n (%)	6/19 (32%)^˦§^	1/9 (11%)	0/12 (0%)	0/11 (0%)	0.03
Severe hypotensive episodes, n (%)	5/19 (26%)	2/9 (22%)	0/12 (0%)	0/11 (0%)	0.08
s/p Septic episode, n (%)	5/19 (26%)	1/9 (11%)	3/12 (25%)	3/11 (27%)	0.81
s/p CPR, n (%)	5/19 (26%)	1/9 (11%)	0/12 (0%)	0/11 (0%)	0.07
Death, n (%)	3/19 (16%)	2/9 (22%)	6/12 (50%)	1/11 (9%)	0.09
Age, n	3	2	6	1	0.08
Mean (SD), mo	28.3 (39.5)	57.7 (24.5)	4.8 (4.6)	0.10	
Median (IQR or min;max), mo	7.8 (3.2;73.8)	57.7 (40.4;75.0)	3.8 (1.5–7.6)	0.10 (-)	

Binary or categorical values given as n/n total (percentage); continuous variables, as mean ± SD and median (IQR or min;max) with min;max indicated in case of n ≤ 3. *P* values (right column) were derived using log-rank, Kruskal–Wallis or chi-squared tests.

*CPR* cardiopulmonary resuscitation, *EBNE* early bilateral nephrectomies, *s/p* state post, *TKV* total kidney volume, *VEBNE* very early bilateral nephrectomies, *VED* very early dialysis.

*P < 0.05 for comparison with VEBNE; **P < 0.01 for comparison with VEBNE; ^#^*P* < 0.05 for comparison with EBNE; ^˦^*P* < 0.05 for comparison with VED; ^˦˦^*P* < 0.01 for comparison with VED; ^§^*P* < 0.05 for comparison with TKV control; ^§§^*P* < 0.01 for comparison with TKV control; ^§§§^*P* < 0.001 for comparison with control.

**Figure 1:**
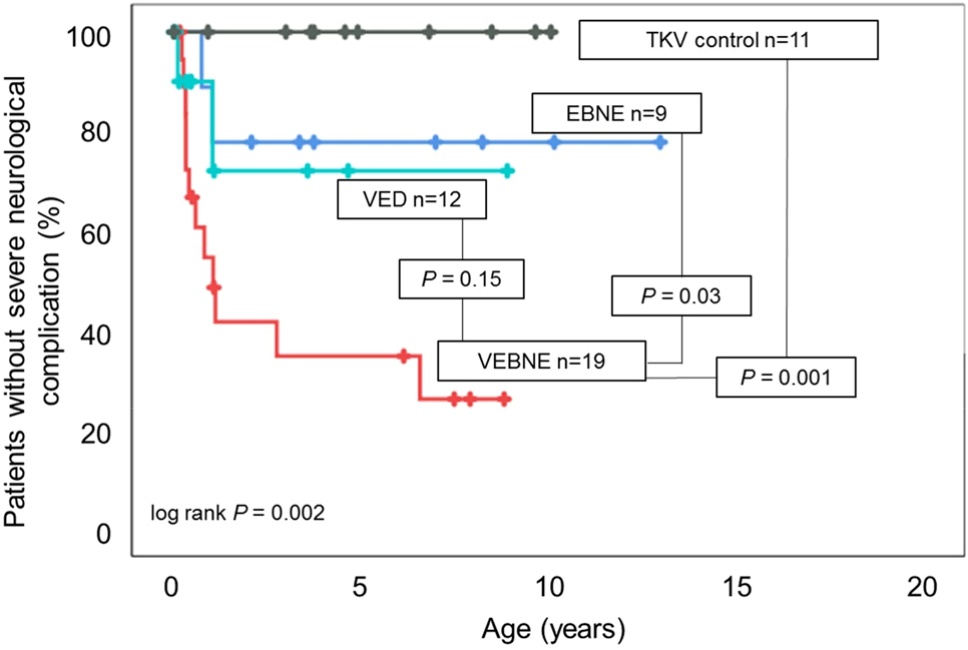
Kaplan–Meier survival without severe neurological complications by patient group. Censored observations (last documented follow-up or death) are marked with a cross. *P* values were derived using log rank tests. *EBNE* early bilateral nephrectomies, *TKV* total kidney volume, *VEBNE* very early bilateral nephrectomies, *VED* very early dialysis.

Follow-up time was similar for VEBNE, EBNE and TKV control patients, but shorter for VED patients (Table 4). Severe neurological complications were further studied over time using Kaplan–Meier analyses and Cox regression analyses. Survival without severe neurological complications after 7 years was approximately 30% in VEBNE, 80% in EBNE, 70% in VED patients and 100% in TKV control patients according to Kaplan–Meier survival analysis (Fig. 1). Thus, VEBNE patients showed significantly worse outcome than EBNE or TKV control patients with a similar trend compared to VED patients. Severe neurological complications in the VEBNE group were evident within the 14 months of life in most cases (10/12). Only in two patients, atrophy of the optic nerve was diagnosed at the ages of 2.8 and 6.6 years.

Overall, severe neurological complications occurred in 16 of 51 (31%) analyzed patients (Table 5). According to Cox regression analysis, the report of severe neurological complications was associated with multiple factors, such as documentation of microcephaly during the course of follow-up as well as weaker and more common neurological symptoms like seizures and reports of severe hypertensive and hypotensive episodes. Furthermore, patients with very early bilateral nephrectomies seemed to be at special risk.

**Table 5:** Cox regression analysis of pre- and postnatal factors associating with severe neurological complications. Cox multiple regression analysis with independent risk factors for severe neurological complications.

	With severe neurological complication (n = 16)	Without severe neurological complication (n = 35)	HR (95% CI)	*P*
**Cox regression analysis**				
*Pre- and perinatal information*				
Oligo- or anhydramnios, n (%)	14/16 (88%)	29/33 (88%)	0.86 (0.20–3.78)	0.84
Prenatal increased echogenicity, n (%)	10/13 (77%)	16/24 (67%)	1.34 (0.37–4.89)	0.66
Prenatal renal hyperplasia, n (%)	13/16 (81%)	17/24 (71%)	1.30 (0.37–4.59)	0.68
Prenatal renal cysts, n (%)	10/14 (71%)	22/29 (76%)	0.76 (0.24–2.44)	0.65
Vaginal delivery, n (%)	8/16 (50%)	13/32 (41%)	1.40 (0.52–3.74)	0.50
Gestational age at birth, mean (SD), weeks	35.1 (2.5) (n = 16)	35.5 (2.4) (n = 30)	0.94 (0.77–1.14)	0.54
Microcephaly at birth, n (%)	1/12 (8%)	1/22 (5%)	1.18 (0.15–9.17)	0.87
Head circumference (Percentile Fenton), mean (SD)	68 (29) (n = 11)	64 (26) (n = 21)	1.01 (0.98–1.03)	0.70
Poor postnatal adaptation, n (%)	12/16 (75%)	23/34 (68%)	1.35 (0.44–4.20)	0.60
Assisted breathing and/or ventilation, n (%)	15/16 (94%)	25/34 (74%)	4.00 (0.53–30.30)	0.18
*Follow-up—complications*				
Seizures, n (%)	14/16 (88%)	9/35 (26%)	12.29 (2.76–54.65)	0.001
Documentation of microcephaly (< P3), n (%)	11/16 (69%)	6/35 (17%)	4.71 (1.63–13.61)	0.004
Severe hypertensive episodes, n (%)	5/16 (31%)	2/35 (6%)	3.12 (1.08–9.05)	0.04
Severe hypotensive episodes, n (%)	7/16 (44%)	0/35 (0%)	13.92 (4.22–45.87)	< 0.001
s/p Septic episode, n (%)	4/16 (25%)	8/35 (23%)	0.89 (0.29–2.76)	0.84
s/p CPR, n (%)	3/16 (19%)	3/35 (9%)	1.60 (0.45–5.62)	0.47
*Patient groups*				
VEBNE, n (%)	12/16 (75%)	7/35 (20%)	6.28 (2.02–19.57)	0.002
EBNE, n (%)	2/16 (13%)	7/35 (20%)	0.46 (0.10–2.01)	0.30
VED, n (%)	2/16 (13%)	10/35 (29%)	0.76 (0.17–3.35)	0.72
TKV control, n (%)	0/16 (0%)	11/35 (31%)	0.03 (0.00–3.07)	0.14
**Cox multiple regression analysis**				
Severe hypotensive episodes, n (%)	7/16 (44%)	0/35 (0%)	11.42 (3.21–40.71)	< 0.001
VEBNE, n (%)	12/16 (75%)	7/35 (20%)	5.12 (1.60–16.39)	0.006

*P* values (right column) were derived from Cox single-variable regression analysis or Cox multiple regression analysis. Binary or categorical values given as n/n total (percentage); continuous variables, as mean ± SD.

*CPR* cardiopulmonary resuscitation, *EBNE* early bilateral nephrectomies, *HR* Hazard Ratio, *s/p* state post, *TKV* total kidney volume, *VEBNE* very early bilateral nephrectomies, *VED* very early dialysis.

To identify independent risk factors associated with severe neurological complications, multivariate Cox regression analysis with consecutive backward selection was performed (including seizures, documentation of microcephaly, severe hypertensive episodes, severe hypotensive episodes and VEBNE), which revealed the report of a severe hypotensive episode and very early bilateral nephrectomies to be independent risk factors for severe neurological complications (Table 5). In this multivariate analysis, patients with very early bilateral nephrectomies were at fivefold elevated risk for severe neurological complications.

### Deaths

Three VEBNE patients died due to cerebral ischemia (age 3.2 months), respiratory failure (7.8 months) and massive postoperative bleeding after revision of dialysis catheter (age 73.8 months). Two EBNE patients died, one due to a septic episode in leukemia (age 40.4 months) and the other patient due to aspiration (75.0 months). Causes of death in six VED patients were indicated as sepsis (ages 5.4 months, 5.7 months, 13.3 months), lung hemorrhage (age 1.8 months) and parental wish to withdraw care in two cases (ages 0.8 months, 2.2 months). Importantly, four of the six deceased VED patients did not display major or severe neurological complications before death. Three out of the four VED patients that passed away without withdrawal of care died after the fifth month of life. One patient from the TKV control group died shortly postnatally due to respiratory failure.

## Discussion

The management of severely affected neonates and infants with ARPKD remains a matter of intense debate. The aim of this study was to examine the association of the timing of bilateral nephrectomies with long-term complications in ARPKD patients.

In our analysis, ARPKD patients with very early bilateral nephrectomies (VEBNE) within the first 3 months of life showed significantly more neurological complications than patients who underwent bilateral nephrectomies receiving their second nephrectomy after the third month of life (EBNE). The severe neurological anomalies observed in VEBNE patients encompassed cerebral ischemia or infarcts, parenchymal brain defects, brain volume decrease, subdural bleeding and (anterior ischemic) optic neuropathy. These complications occurred predominantly during the first year of life.

The rationale of drawing a line for evaluation of consequences of bilateral nephrectomies at the age of 3 months was based on two considerations: firstly, major maturation processes of autonomic cardiovascular control seem to occur within the first 3 months of life^19,20^. Animal models suggest that renal sympathetic nerve control matures postnatally^21^. Secondly, both plasma renin activities and aldosterone levels^22,23^ have been reported to peak within the first 3 months with a steep decline afterwards. Data from healthy infants demonstrated a remarkable increase of systolic blood pressure during the first 6 weeks and a physiologic dip of diastolic blood pressure after birth with a gradual increase during the first year of life from month two to three on^24^. Summarizing these hypotheses, the removal of both kidneys in this early phase of life might result in the maximum impact by abolishing blood pressure autoregulation. Plotting the occurrence of severe neurological complications against the age at second nephrectomy supports the choice of drawing a line at the age of 3 months as critical time point (Suppl. Figure S1).

Interestingly, our analysis demonstrated that VEBNE and severe hypotensive episodes were independently associated with severe neurological complications. In addition to VEBNE additional factors leading to hypotensive episodes seem to be involved, e.g. acute illnesses^25^. Previous smaller case series reported an anephric status and ARPKD as risk factors for development of anterior ischemic optic neuropathy in patients on PD^26^. Hypovolemia has been reported in half of the affected infants with anterior ischemic optic neuropathy on PD^26^. In our study, optic neuropathy was frequently observed in the VEBNE group. One might speculate about common pathophysiologic mechanisms underlying to all types of cerebral defects, such as reduced cerebral autoregulation and chronic hypotension with increased vulnerability in acute hypotensive episodes. Fluid management per se can pose major challenges in infants on PD, but might affect blood pressure more immediately in anephric infants. In our analysis, we did not have comprehensive data on the fluid status of patients. It is worth noting that cardiopulmonary resuscitation was documented most frequently in the VEBNE patient group. Despite the challenges of fluid management in anephric infants, PD remains the preferred modality for ARPKD infants^11^. A recent analysis showed that technique survival in ARPKD is comparable to that observed in other early-onset kidney diseases, although PD in ARPKD may require smaller fill volumes and/or more cycles^27^. Our analysis emphasizes in particular the association of documented pronounced hypotensive episodes with severe neurological complications in ARPKD patients receiving early bilateral nephrectomies and underlines the recommendation of very tight blood pressure control for all anephric children on PD. Furthermore, measures to avoid hypotensive episodes on PD need to be considered, e.g. by avoiding high glucose solutions or by sodium supplementation in dialysate and nutritionally^25,28,29^. On the other hand, ARPKD specific therapeutic options and clinical studies are eagerly awaited for this severely affected patient population.

None of the TKV control patients with massively enlarged kidneys developed severe neurological complications. Caution is required not to anticipate postnatal severe courses in all cases of newborns with pre- and perinatal anomalies^30^.

Severe neurological complications were observed in two VED patients: in one patient cerebral ischemia occurred during a very difficult postnatal adaptation requiring intense cardiorespiratory support and unilateral nephrectomy. This patient passed away at the age of 4 weeks after withdrawal of intensive care treatment upon parental request. The other patient died from sepsis at 13 months of age after documentation of hydrocephalus with brain atrophy 1 month earlier. All four of the six deceased VED infants who did not experience severe neurological complications deceased within the first 8 months of life. This is important for the interpretation of the Kaplan–Meier survival analysis for severe neurological complications (Fig. 1). Due to their early death, these patients were no longer at risk for severe neurological complications. Thus, death could be a competing risk for severe neurological complications in this specific subcohort. Survival without severe neurological complications tended to be better in VED compared to VEBNE patients, although statistical significance was missed due to small sample size. Importantly, the majority of the deceased VED patients died well beyond 3 months of age. EBNE patients were also severely affected with similar Apgar values as VEBNE and early dialysis dependency. Yet, only two of nine EBNE patients suffered from severe neurological sequelae, arguing against an intrinsic ARPKD-specific neurologic phenotype.

The current study faces limitations: as severely affected and/or early deceased patients are likely to be underrepresented in our registry study, the obtained sample sizes of the four groups are rather small. Furthermore, there might be a center bias in deciding for nephrectomies in infants. The risk factor severe hypotensive episodes needs to be interpreted carefully: the report and documentation might be biased by the personal perception of the attending physicians and severely affected children might spend more time in hospital, where blood pressure documentation could reveal anomalies more frequently. Hypotensive episodes are known to frequently occur within hypovolemia in infants on PD^25,31,32^. Our study describes interesting associations of severe hypotensive episodes and the decision to perform VEBNE with severe neurological complications, but we cannot prove causality due to the retrospective registry data underlying the analysis. Importantly, in severely affected infants there may be situations where the decision for uni- or bilateral nephrectomies appears inevitable. Given our data a thorough discussion of all relevant aspects both with the parents and the attending medical team seems important.

## Conclusion

Neurological complications occur more frequently in patients with bilateral nephrectomies performed during the first 3 months of life compared to patients with the second nephrectomy performed between 4 and 15 months of age. Our data suggest utmost caution in the decision-making process for very early bilateral nephrectomies, especially in infants with partially conserved residual renal function, and support postponing the second nephrectomy when possible. Importantly, every effort should be made to avoid pronounced hypotensive episodes in this population.

## Methods

### Registry

The international cohort study ARegPKD follows patients with the clinical diagnosis of ARPKD according to the previously described protocol^17,18^. In summary, basic data and regular follow-up data sets are obtained and are subject to regular data quality control. The study protocol was approved by the Ethics Committee of the Faculty of Medicine of Cologne University and the Institutional Review Boards of participating sites. Informed consent was obtained from all subjects or, if subjects are under 18, from a parent and/or legal guardian according to applicable local regulations. All methods were carried out in accordance with relevant guidelines and regulations.

For this specific analysis ischemic brain defects, hypoxic brain damage, brain infarct, parenchymal defect, severe neurodevelopmental disorder and optic neuropathy with vision loss were rated as severe neurological complication. For detailed classification of neurocognitive development, we re-surveyed data on head circumferences at birth and during further course, school education, specific therapies, developmental milestones and psychological/intelligence testing for the studied subcohort: psychomotoric development was classified as normal or mildly, severely or very severely disordered. Head circumferences at birth were graded in percentiles according to the revised Fenton growth chart^33^.

All *PKHD1* variants were classified according to criteria of the American College of Medical Genetics (ACMG)^34^. Sonography-based kidney volumes were calculated according to the ellipsoid formula (length × width × depth × π/6) and standardized to standard deviation scores (SDS) to pediatric normal values^35,36^.

### Statistics

Data analysis was performed on the dataset available in September 2018 using SPSS 25 (IBM Corp., Armonk, NY, USA) for statistical analyses. Data completeness varied by variable. Continuous variables were described using the number of non-missing values, mean and standard deviation (SD) as well as median and interquartile range (IQR). For binary or categorical variables, absolute and relative frequencies were provided. Event-free survival rates were estimated by the Kaplan–Meier method. Follow-up duration in the four groups were calculated using the reverse Kaplan–Meier method, displayed as median (IQR) and compared using log-rank test. Differences of (continuous) sonographic parameters between VEBNE and control group were compared by Mann–Whitney U test. Differences between the four patient groups were assessed by Kruskal–Wallis tests for continuous and by chi-squared tests for binary or categorical variables. The statistical tests did not adjust formally for multiplicity due to the exploratory nature of the analysis. No imputation was performed.

To investigate the predictive value of clinical risk factors for severe neurological outcome, Cox regression analysis was performed for each risk factor separately. The parameters with a *p* value below 0.10 were then included in a Cox multiple regression analysis with consecutive backward selection for variables with a *p* value below 0.10.

To assess the differences of survival without severe neurological complications in the four patient groups, Kaplan–Meier analysis and log rank tests were used. All analyses are exploratory and p values of less than 0.05 were considered significant in a descriptive manner in distinguishing between the groups.

## Supplementary information

Supplementary file1

## Data Availability

The data that support the findings of this study are available, on reasonable request, from the corresponding author.

## Abbreviations

ARPKD: Autosomal recessive polycystic kidney disease
CLKTx: Combined liver and kidney transplantation
CVVH: Continuous veno-venous hemofiltration
EBNE: Early bilateral nephrectomies
FTT: Failure to thrive
HR: Hazard ratio
HD: Hemodialysis
KRT: Kidney replacement therapy
NE: Nephrectomy/nephrectomies
P: Percentile
PD: Peritoneal dialysis
PTP: Pole-to-pole
SDS: Standard deviation scores
TKV: Total kidney volume
VEBNE: Very early bilateral nephrectomies
VED: Very early dialysis
