# Supplementary material for: Severe neurological outcomes after very early bilateral nephrectomies in patients with autosomal recessive polycystic kidney disease (ARPKD)
Source: Sci Rep. 2020 Sep 29;10:16025. doi: 10.1038/s41598-020-71956-1 (PMC7525474; doi:10.1038/s41598-020-71956-1)

**Supplementary Information of the Manuscript**

**Severe neurological outcomes after very early bilateral nephrectomies in patients with autosomal recessive polycystic kidney disease (ARPKD)**

Kathrin Burgmaier MD, Gema Ariceta MD, Martin Bald MD, Anja Katrin Buescher MD, Mathias Burgmaier MD, PhD, Florian Erger MD, Michaela Gessner MD, Ibrahim Gokce MD, Jens König MD, Claudia Kowalewska MD, Laura Massella MD, Antonio Mastrangelo MD, Djalila Mekahli MD, PhD, Lars Pape MD, Ludwig Patzer MD, Alexandra Potemkina MD, Gesa Schalk MD, Raphael Schild MD, Rukshana Shroff MD, PhD, Maria Szczepanska MD, Katarzyna Taranta-Janusz MD, PhD, Marcin Tkaczyk MD, PhD, Lutz Thorsten Weber MD, Elke Wühl MD, Donald Wurm MD, Simone Wygoda MD, Ilona Zagozdzon PhD, Jörg Dötsch MD, Jun Oh MD, Franz Schaefer MD, Max Christoph Liebau MD & the ARegPKD consortium

**Suppl. Figure 1. Association of age at second nephrectomy and neurological complications.**

Plot of occurrence of severe neurological complications (A) or grade of psychomotoric development (B) against age at second nephrectomy in 19 VEBNE and 9 EBNE patients.Data regarding psychomotoric development (B) based on a re-survey and were available for 17 VEBNE and 8 EBNE patients. EBNE: early bilateral nephrectomies, VEBNE: very early bilateral nephrectomies.

**Suppl. Figure 1:**


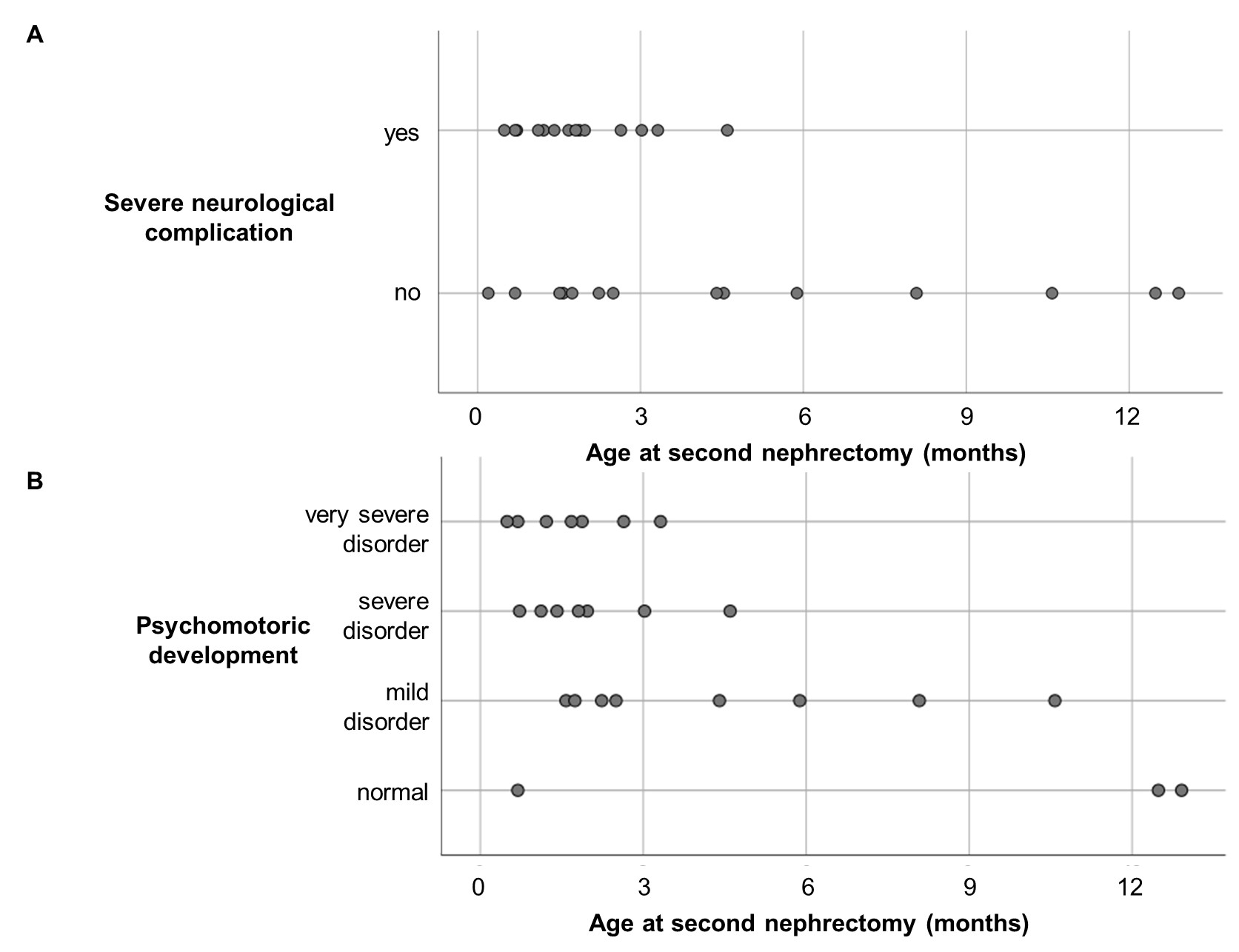

Supplement: Supplementary file 1 — Supplementary file1 [file 41598_2020_71956_MOESM1_ESM.docx]
